# Supplementary material for: Mixology: a tool for calculating required masses and volumes for laboratory solutions
Source: Wellcome Open Res. 2021 May 26;6:128. [Version 1] doi: 10.12688/wellcomeopenres.16617.1 (PMC8185578; doi:10.12688/wellcomeopenres.16617.1)
Supplement: Peer review history

# Peer review: Reviewer response for version 1

**DOI:** 10.21956/wellcomeopenres.18319.r44076

**Published:** 2021-06-07

**Authors:** Pablo Cárdenas

**Affiliations:** 1 Department of Biological Engineering, Massachusetts Institute of Technology, Cambridge, MA, USA

This article describes "Mixology", a novel software tool used for preparing chemical solutions used in laboratory science. Given a list of user-defined reagents with their initial and desired concentrations, the software automates calculations for quantity conversion of different molar amounts, activities, masses, and volumes required to prepare a specified amount of solution. The software automatically retrieves any relevant molecular weights of reagents for the calculations. This not only saves time for researchers preparing experiments but more importantly, can reduce mistakes committed when doing the calculations by hand, as explained by the author. Mixology is free and open source, simple in design and use, and quickly accessible across platforms as an online application. Because of these reasons, it has the potential to be a very useful tool for wet lab scientists accessing it from a variety of devices, both in and out of the lab.

 I have no comments on the article text. Below, I will offer some potentially useful feedback on the web app itself:

**Design and functionality:**

- Although the web app is very easy to use once I understood exactly what it does and how it works, this was not immediately evident to me. This is clearly explained in the video linked under the expandable "About" section, as well as on the animated GIF file on the GitHub README (which I thought was particularly useful, since I found it to be quicker than the full video). However, the simplest way I can think of to show what the app does is to have an "example" button on the bottom row of links that opens the permalink to an example solution for the user to play with, maybe the one displayed on Figure 2.
- The permalink function is an excellent idea, as it allows users to easily store and share recipes. However, some users might prefer (or might be required) to copy the full recipe onto their own laboratory notebooks, whether electronic or physical. A drawback of Mixology's excellent dynamically-updated user interface is that it does not allow for easy copy-paste of text off of the app. Adding a function to export recipes as plain text would be very useful.
- If not too involved, it could be intuitive to allow users to click on the grey "(MW: XX.XXXX)" text that appears to the right of a chemical reagent's name as an additional shortcut to the " Custom molecular mass: " pop-up.
- This is absolutely trivial, but adding uppercase L as an alternative symbol for liters (as well as mL, µL, etc.) would be appreciated for aesthetic reasons and muscle memory.

**Performance:**

 I can confirm the app performs well on Firefox (v.89.0), Chrome (v.91.0.4472.77), and Safari (v.14.1.1) for macOS Big Sur 11.4 with US keyboard encodings. I was not able to get it to work with any mobile browsers on iOS and iPadOS 14.4.2, where the app freezes once I click the "add a compound button", an error also reported by other reviewers for Safari v.14.0 on macOS v.10.15.7. Fortunately, the issue was solved after updating to iOS 14.6, and I suspect the desktop Safari issue would be solved in a similar fashion. I was not able to replicate and trace the original bug on mobile debuggers and emulators, but I would expect it to be related to the software's dependencies rather than the code presented here. I was not able to test the app's performance on Android-based mobile operating systems.

 In this light, perhaps the "About" section could include a reminder for users to update their operating system if they run into trouble. I can envision mobile access to be one of the main usage contexts for the app, especially in lab spaces without a computer on the bench. I realize this may not be the original application context envisioned for the app, as I found a warning message displaying "mixology is easier to use with a wider screen" to be triggered by mobile browsers in the source code (incidentally, this warning did not trigger on my mobile devices).

 Overall, I very much value the work presented by the author and will surely be using it in my own work.

Are the conclusions about the tool and its performance adequately supported by the findings presented in the article?

Yes

Is the rationale for developing the new software tool clearly explained?

Yes

Is the description of the software tool technically sound?

Yes

Are sufficient details of the code, methods and analysis (if applicable) provided to allow replication of the software development and its use by others?

Yes

Is sufficient information provided to allow interpretation of the expected output datasets and any results generated using the tool?

Yes

Reviewer Expertise:

Wet lab molecular and cell biology, computational biology, scientific web application development

I confirm that I have read this submission and believe that I have an appropriate level of expertise to confirm that it is of an acceptable scientific standard.

# Peer review: Reviewer response for version 1

**DOI:** 10.21956/wellcomeopenres.18319.r44070

**Published:** 2021-06-01

**Authors:** Joachim Goedhart

**Affiliations:** 1 Molecular Cytology, Swammerdam Institute for Life Sciences, van Leeuwenhoek Centre for Advanced Microscopy, University of Amsterdam, Amsterdam, The Netherlands

The paper reports on a new, freely available and open-source web app 'Mixology' that simplifies the calculations necessary for preparing solutions that are used in labs. The advantages of using an app over manual calculations are clearly explained in the paper.

 I have reviewed the manuscript and the app, but I haven't reviewed the source code (as the coding language that is used is outside my area of expertise).

**Comments on the manuscript:**

- The example calculation in the paper (11.2 ml) and the screenshot of the app (figure 1, 11.41 ml) show slightly different numbers (probably due to rounding at step 4). I'd recommend to make the numbers consistent.
- Using the same abbreviation for litre (uppercase L or lowercase l) improves consistency.
- The paper states "Concentrations can be entered in any of several unit forms", including µg/ml, but the app seems unable to accept the greek symbol 'mu' (see also comments below). If this is a limitation of the app, it should be highlighted in the paper.
- The bullet point "mass-based: g/ml, µg/ml, mg/l, % (w/v), etc." appears twice.

**Comments on the webtool:**

- The webtool can be used without reading the paper, suggesting that the user interface is intuitive.
- The automatic retrieval of molecular weights is elegant and works smoothly.
- The ability to generate a permalink is fantastic and will simplify the sharing of recipes.
- The app didn't work for me on Safari (version 14.0, on Mac OS version 10.15.7) as I was unable to add compounds (for unclear reasons). It worked well on Firefox (version 88.0.1) and a mobile device.
- I was able to reproduce a buffer that we use for imaging and the quantities that we normally use match with the results of the app. I was able to generate a permalink for the recipe (https://mixology.science/?recipe=MpW9AwsswL).
- When I generate or retrieve the permalink, the decimal separator becomes a comma instead of a period. This turned out to be due to the language setting in Firefox (Dutch) and was solved by setting the language to English.
- Accepting the micro (µ) sign in addition to the u would be an improvement.
- The simplicity of the app is a strength, but one can easily think of features that could be added. Some improvements that I can think of:
  1. Having grams as a default, instead of having to type it for each line
  2. Showing the options of 'conc. unit' and 'mass unit' in a dropdown menu (or as tooltip)
  3. Option to copy the permalink to the clipboard (or copy it to clipboard at the time it is generated). This can be another button 'copy permalink'.
  Ultimately, the design of the app (and choice of which features to implement) should be the choice of the author(s).
- The author invites readers to add suggestions to the Github repo and I would suggest to include the link to the repo immediately after that statement.
- Does the author also accept comment requests through other channels (email, twitter) for readers not familiar with Github? If so, it would be helpful to indicate that.

Are the conclusions about the tool and its performance adequately supported by the findings presented in the article?

Yes

Is the rationale for developing the new software tool clearly explained?

Yes

Is the description of the software tool technically sound?

Yes

Are sufficient details of the code, methods and analysis (if applicable) provided to allow replication of the software development and its use by others?

Yes

Is sufficient information provided to allow interpretation of the expected output datasets and any results generated using the tool?

Yes

Reviewer Expertise:

Cell biology, Data visualization, Web apps.

I confirm that I have read this submission and believe that I have an appropriate level of expertise to confirm that it is of an acceptable scientific standard.
